# Higher Viral Load of Emerging Norovirus GII.P16-GII.2 than Pandemic GII.4 and Epidemic GII.17, Hong Kong, China

**DOI:** 10.3201/eid2501.180395

**Published:** 2019-01

**Authors:** Sarah K.C. Cheung, Kirsty Kwok, Lin-Yao Zhang, Kirran N. Mohammad, Grace C.Y. Lui, Nelson Lee, E. Anthony S. Nelson, Raymond W.M. Lai, Ting F. Leung, Paul K.S. Chan, Martin Chi-Wai Chan

**Affiliations:** The Chinese University of Hong Kong, Hong Kong, China

**Keywords:** emerging GII.P16-GII.2, epidemic GII.17, gastroenteritis, norovirus, pandemic GII.4, recombination, viral load, viruses, Hong Kong, China

## Abstract

We compared viral load of emerging recombinant norovirus GII.P16-GII.2 with those for pandemic GII.Pe-GII.4 and epidemic GII.P17-GII.17 genotypes among inpatients in Hong Kong. Viral load of GII.P16-GII.2 was higher than those for other genotypes in different age groups. GII.P16-GII.2 is as replication competent as the pandemic genotype, explaining its high transmissibility and widespread circulation.

Norovirus, the leading cause of acute gastroenteritis, evolves through mutation and recombination (*1*). Noroviruses are named by dual nomenclature using the genotype of RNA-dependent RNA-polymerase (RdRp) and major capsid protein (VP1) (*2*). Recently, 2 recombinant noroviruses carrying RdRp genotype GII.P16 with 2 other VP1 genotypes emerged and spread worldwide: GII.P16-GII.4 in the United States and Europe and GII.P16-GII.2 in Europe and Asia in 2016 (*3**–**5*). GII.P16 actively recombined with >8 capsid genotypes (*6**–**8*) and may have pandemic potential and lead to a change in norovirus epidemiology. Phylogenetic and sequence analyses indicated that recent GII.P16-GII.2 had no remarkable change on capsid protein compared with earlier GII.2 strains, suggesting that factors other than immune escape or change in affinity for histo–blood group antigens (i.e., host susceptibility) may play a role in the recent reemergence (*6*). We compared the viral load of norovirus GII.P16-GII.2 with pandemic GII.Pe-GII.4 and epidemic GII.P17-GII.17 in a cohort of hospitalized patients in Hong Kong over a 5-year period. Our findings may explain, at least in part, the high transmissibility and widespread circulation of GII.P16-GII.2.

## The Study

This study was part of an ongoing molecular surveillance study of norovirus genotype in hospitalized cases in Prince of Wales Hospital, Hong Kong, during August 2012–June 2017. Norovirus genotype distribution has been detailed in earlier reports (*9**,**10*). The fecal norovirus load was determined by a genogroup-specific quantitative real-time reverse transcription PCR (qRT-PCR) assay (*11*) (Appendix) and was expressed as cycle threshold (C_t_) value that has been demonstrated in a large-scale analysis of CaliciNet data to associate with host and virologic factors (*12*). A lower C_t_ value represents a higher norovirus load (Appendix Figure 1). In the data analysis, we stratified cases by 3 patient age groups: <5 years, 5–65 years, and >65 years. Continuous variables between 2 and 3 groups were compared by the Mann-Whitney U test and the Kruskal-Wallis test with Dunn’s multiple comparison correction, respectively, by Prism 7 for Mac (GraphPad, https://www.graphpad.com/scientific-software/prism). A 2-tailed p value <0.05 was considered statistically significant.

During the 5-year period, we collected fecal samples at admission from 1,465 hospitalized patients with laboratory-confirmed norovirus gastroenteritis. The median age of patients was 3 years (interquartile range [IQR] 1–50 years); male:female ratio was 1:1.1. Norovirus genotype was successfully determined for 1,269 (86.6%) samples. We excluded 8 (0.6%) patients co-infected with >1 norovirus genotype from viral load analysis. The top 3 circulating norovirus genotypes were GII.Pe-GII.4 (n = 657; 51.8%), GII.P17-GII.17 (n = 191; 15.1%), and GII.P16-GII.2 (n = 136; 10.7%).

We found that the viral load was higher for emerging GII.P16-GII.2 norovirus than for pandemic GII.Pe-GII.4 and epidemic GII.P17-GII.17 (Figure, panel A). In young children <5 years of age, the median viral load of GII.P16-GII.2 was as high as that of GII.Pe-GII.4 (median C_t_ [IQR]: GII.P16-GII.2, 15.2 [12.9–18.8]; GII.Pe-GII.4, 16.7 [14.8–19.0]; p = 0.200). In patients 5–65 years of age, the median viral load of GII.P16-GII.2 was 28-fold higher than that of GII.Pe-GII.4 and 42-fold higher than that of GII.P17-GII.17. In patients >65 years of age, the median viral load of GII.P16-GII.2 was 45-fold higher than that of GII.Pe-GII.4 and 274-fold higher than that of GII.P17-GII.17. The median viral load of GII.Pe-GII.4 declined with age, whereas that of GII.P16-GII.2 remained at the same high level among different age groups. GII.P17-GII.17 had the lowest viral load in most comparisons.

**Figure:**
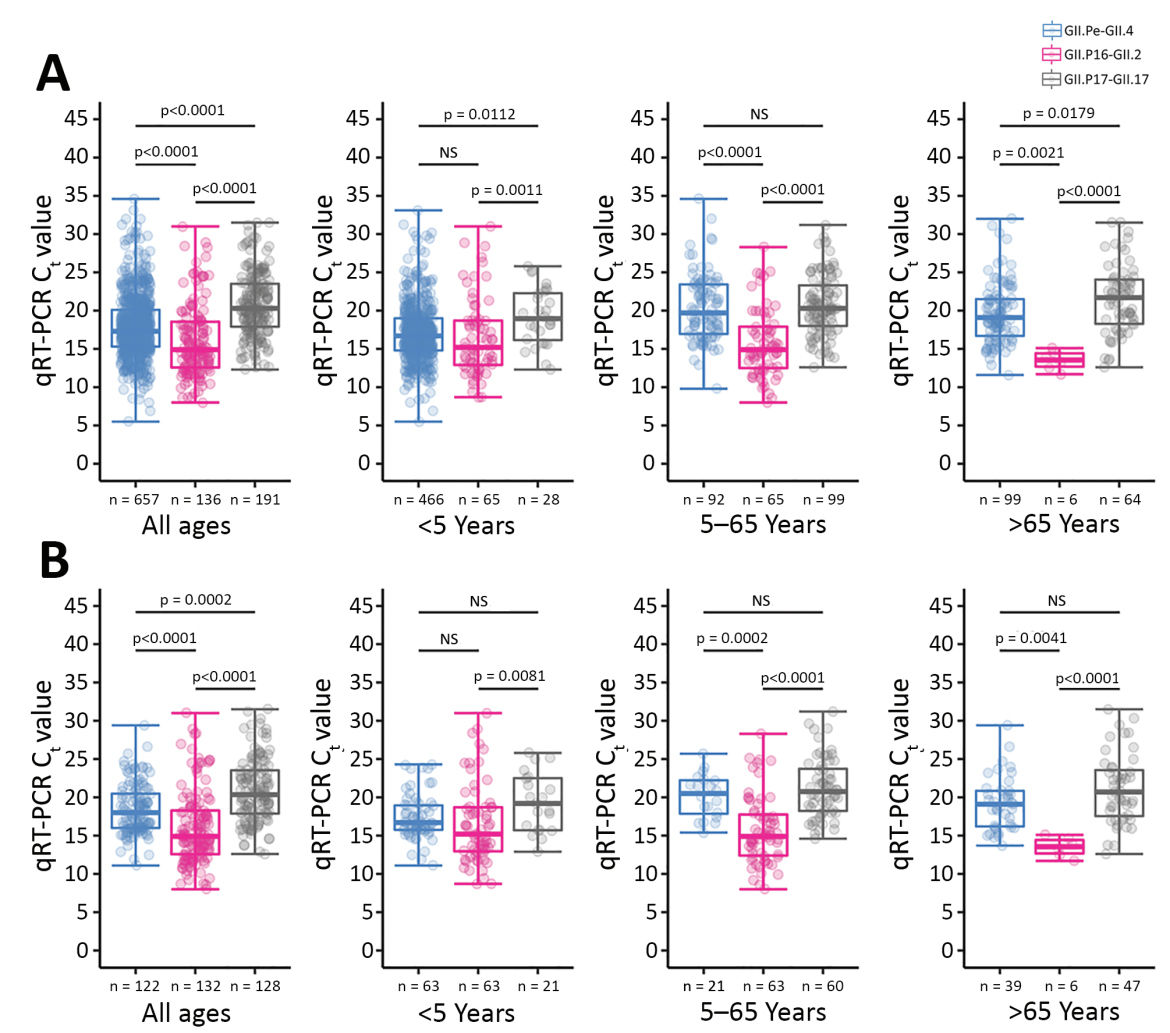
Higher fecal viral load of recombinant norovirus genotype GII.P16-GII.2 compared with pandemic GII.Pe-GII.4 and epidemic GII.P17-GII.17 among patients in Hong Kong, August 2012–June 2017. A) Results for the whole study period; B) results for the first season of emergence for each genotype: GII.Pe-GII.4, August 2012–June 2013; GII.P16-GII.2, July 2016–June 2017; and GII.P17-GII.17, July 2014–June 2015. Data shown are stratified by age group of patients. Each dot represents a patient; box tops and bottoms indicate interquartile range; horizontal lines within boxes indicate medians; error bars indicate maxima and minima. C_t_ values were determined by qRT-PCR and used as proxies for norovirus load. A lower C_t_ value indicates a higher norovirus load. p values were calculated by the Kruskal-Wallis test, with Dunn’s multiple comparison corrections. C_t_, cycle threshold; NS, not significant; qRT-PCR, quantitative reverse transcription PCR.

We did 2 additional subgroup analyses (sensitivity tests) to validate the robustness of the high viral load observation of GII.P16-GII.2. First, we compared the viral load of cases only during their first season of emergence (i.e., in an immune naive population): for GII.Pe-GII.4, August 2012–June 2013; for GII.P17-GII.17, July 2014–June 2015; and for GII.P16–GII.2, July 2016–June 2017. We observed a trend similar to that of all cases in which viral load of GII.P16-GII.2 was as high as GII.Pe-GII.4 in young children <5 years of age and higher than those of GII.Pe-GII.4 and GII.P17-GII.17 in older children, adults, and the elderly (Figure, panel B). Second, we compared co-circulating GII.P16-GII.2 and GII.Pe-GII.4 in the last season, July 2016–June 2017, to minimize sample processing variation over time. Again, we observed a trend similar to that of all cases and those during first season of emergence (Appendix Figure 2).

To validate the robustness of C_t_ values, we randomly selected 80 samples (16 samples/season) according to quality control sampling scheme ANSI/ASQ Standard Z1.4 (https://asq.org/) for repeat qRT-PCR measurement and inhibition study. We found a strong association between initial and repeat measurements (Spearman r = 0.82; p<0.0001) (Appendix Figure 3). Most samples gave an ideal C_t_ difference of ≈1 between undiluted and 2-fold diluted templates (median C_t_ difference [IQR] 1.0 [0.9–1.1]), indicating minimal to mild inhibition (Appendix Figure 4). To exclude the possibility of genotype-specific quantification artifacts, we inspected the amplification efficiency of the assay by testing on 5-fold serial dilution of 3 strains for each virus genotype. The qRT-PCR efficiency in GII.Pe-GII.4 (100.8 ± 5.3%) and GII.P17-GII.17 (99.1 ± 2.6%) was equivalent to that of GII.P16-GII.2 (95.0 ± 4.3%) (p = 0.296 by 1-way ANOVA). We randomly selected 13 of 72 samples with low viral load (C_t_ >25.0) for primers/probe sequence mismatch analysis; mismatch was noted in only 1 case, indicating that >96% samples with low viral load were free of primers/probe mismatch.

## Conclusions

We found that GII.P16-GII.2 shed in higher amounts than pandemic GII.Pe-GII.4 in different age groups. This new strain, which is as replication competent as pandemic GII.Pe-GII.4, may cause severe gastroenteritis and lead to poor clinical outcomes (*13*). Our findings imply that the absence of prior exposure to this newly emerged strain may result in the delayed immune response and viral clearance in most populations. Immune naivety may be attributed to equally high viral loads of GII.P16-GII.2 and GII.Pe-GII.4 in children (*1*), providing a virologic explanation for the recent upsurge in the number of outbreaks caused by GII.P16-GII.2 in nursery schools, kindergartens, and elementary schools in Japan in the winter of 2016–17 (*14*). That report found a higher reproductive number of GII.P16-GII.2 compared with the previous 4 seasons, during which other norovirus genotypes, such as GII.Pe-GII.4, predominated, a result consistent with our findings of prominent viral load of GII.P16-GII.2 in children.

Our findings agree with a previous phylogenetic analysis, which showed that the capsid of GII.P16-GII.2 was closely related to earlier GII.2 strains, when it was speculated that its recent emergence may be attributable to high replication efficiency (*6*). Furthermore, recombinants carrying GII.P16, including GII.P16-GII.4 and GII.P16-GII.2, have caused >60% of norovirus outbreaks in 2016 and 2017 in the United States (CaliciNet, https://www.cdc.gov/norovirus/reporting/calicinet/data.html). We propose that this time, rather than acquiring a new capsid variant, a new polymerase variant GII.P16 may be affecting norovirus epidemiology worldwide. This emerging and actively recombining norovirus polymerase genotype GII.P16 is highly transmissible, with pandemic risk. The mechanism behind replication difference among norovirus genotypes needs to be further studied by a virus cultivation system such as human intestinal enteroids (*15*).

Our study has limitations. First, we did not evaluate the viral load of GII.P16-GII.4 because this recombinant was sporadically (n = 21; 1.7%) observed. Second, we did not perform multivariate analysis to control for other confounding factors such as time from symptom onset to sample collection (viral load decreases over time) because of incomplete information, which may have introduced bias.

In summary, our results show that the emerging recombinant norovirus GII.P16-GII.2 is as replication competent as pandemic genotypes, which explains its high transmissibility and widespread circulation. Norovirus GII.P16-GII.2 has pandemic potential.

Appendix Discussion of materials and methods used for the study of norovirus GII.P16-GII.2.
