# Supplementary material for: Higher Viral Load of Emerging Norovirus GII.P16-GII.2 than Pandemic GII.4 and Epidemic GII.17, Hong Kong, China
Source: Emerg Infect Dis. 2019 Jan;25(1):119–22. doi: 10.3201/eid2501.180395 (PMC6302574; doi:10.3201/eid2501.180395)
Supplement: Appendix — Discussion of materials and methods used for the study of norovirus GII.P16-GII.2. [file 18-0395-Techapp-s1.pdf]

Article DOI: https://doi.org/10.3201/eid2501.180395

Higher Viral Load of Emerging Norovirus GII.P16-GII.2 than Pandemic GII.4 and Epidemic GII.17, Hong Kong, China

Appendix

Materials and Methods

Measurement of Norovirus Load by Quantitative Reverse Transcription PCR

We prepared a 10% (wt/vol) fecal matter suspension in 0.85% saline. After centrifugation, we extracted RNA from 200 μL of supernatant using a MagMAX automation system and MagMAX Viral RNA isolation kit (ThermoFisher Scientific, Waltham, MA, USA). RNAs were eluted in 50 μL of the elution buffer provided. We included norovirus-positive and -negative controls in each extraction run. We quantified norovirus RNA by using the SuperScript III Platinum One-Step qRT-PCR kit (Invitrogen, Carlsbad, CA, USA) or TaqMan Fast Virus 1-Step Master Mix (Applied Biosystems, Foster City, CA) on a 7900HT Fast Real-Time PCR System (Applied Biosystems). We included a prequantified in vitro-transcribed norovirus RNA in each run for standardization. Primers and probe sequences and thermal cycling conditions are as previously described (*1*).

Reference

1. Kageyama T, Kojima S, Shinohara M, Uchida K, Fukushi S, Hoshino FB, et al. Broadly reactive and highly sensitive assay for Norwalk-like viruses based on real-time quantitative reverse transcription-PCR. J Clin Microbiol. 2003;41:1548–57. http://dx.doi.org/10.1128/JCM.41.4.1548-1557.2003

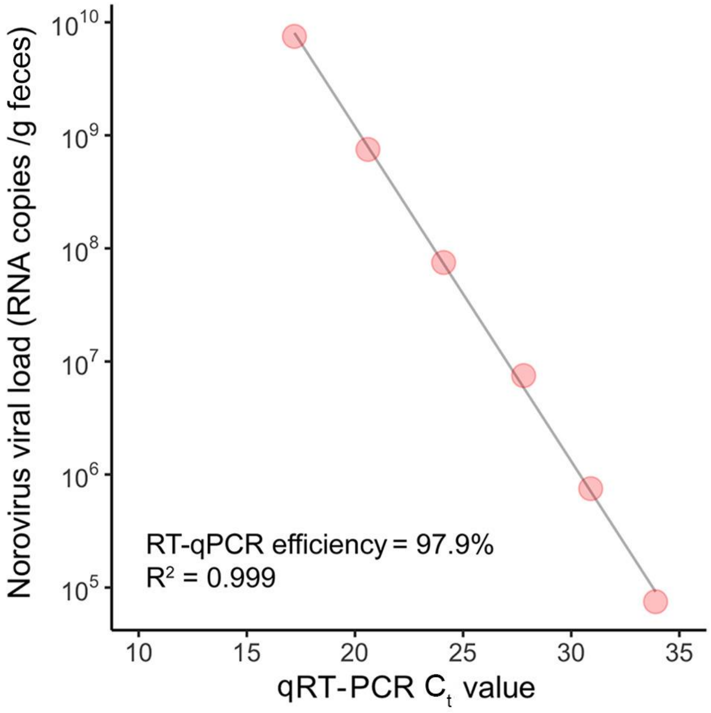

Appendix Figure 1. High linearity between cycle threshold (C_t) values of quantitative reverse transcription PCR (qRT-PCR) and norovirus RNA copy numbers. Shown is 1 representative standard curve of 10-fold serial dilutions of an in vitro-transcribed RNA of norovirus GII with a high amplification efficiency of 97.9% and linearity (R^2) of 0.999.

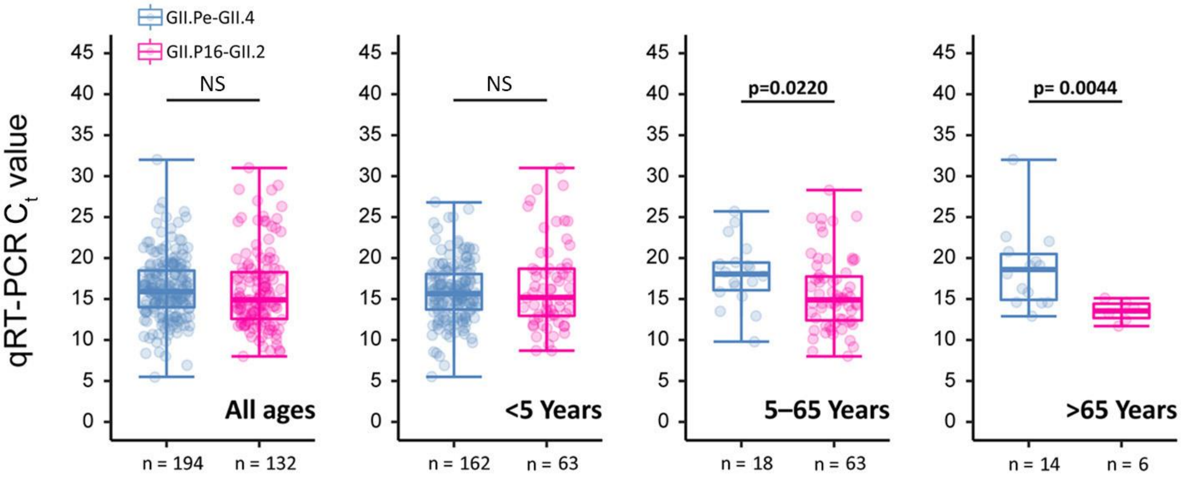

Appendix Figure 2. Higher viral load of recombinant norovirus genotype GII.P16- GII.2 compared with cocirculating pandemic GII.Pe-GII.4 from July 2016 to June 2017. Data shown are stratified by age group of patients: all ages, <5 years, 5–65 years, and >65 years. Cycle threshold (C_t) values were determined by quantitative reverse transcription PCR (qRT-PCR) and used as proxies for norovirus load. A lower C_t

value indicates a higher norovirus load. Each dot represents a patient; box tops and bottoms indicate interquartile range; horizontal lines within boxes indicate medians; error bars indicate maxima and minima. P values were calculated by the Mann-Whitney U-test. NS, not significant.

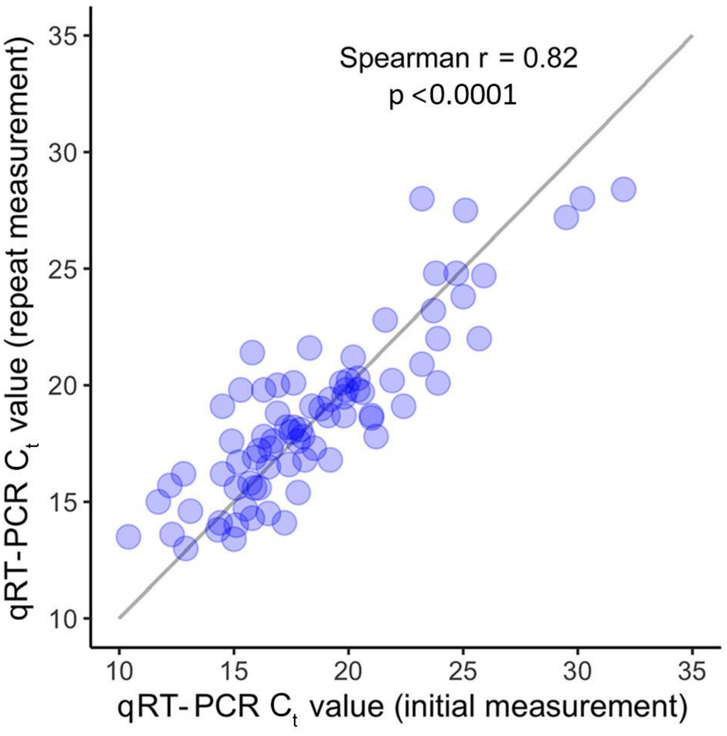

Appendix Figure 3. Highly correlated cycle threshold (C_t) values of quantitative reverse transcription PCR (qRT-PCR) between initial and repeat measurements. A subset of 80 samples (16 samples per season) was randomly selected and tested. The diagonal gray line denotes a hypothetical fit line with a slope of 1 between identical paired measurements.

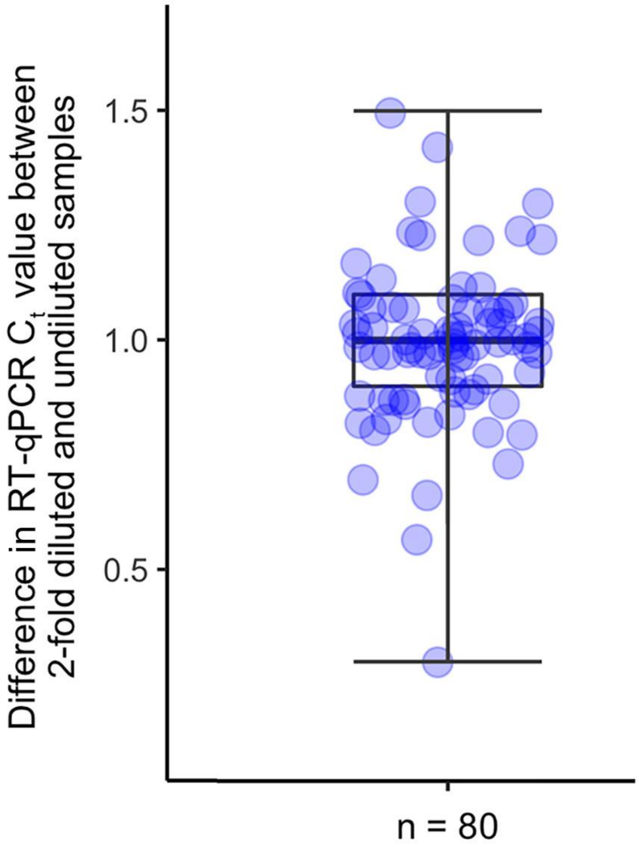

Appendix Figure 4. Presence of minimal-to-mild PCR inhibition in fecal samples. Shown is a box plot of difference in cycle threshold (C_t) values of quantitative reverse transcription PCR (qRT-PCR) between undiluted and 2-fold diluted input viral RNA. A subset of 80 samples (16 samples per season) was randomly selected and tested. The theoretical C_t difference in samples without any qRT-PCR inhibition or enhancement should read as 1.0.
